# Peritoneal Metastasis: Current Status and Treatment Options

**DOI:** 10.3390/cancers14010060

**Published:** 2021-12-23

**Authors:** Lilian Roth, Linda Russo, Sima Ulugoel, Rafael Freire dos Santos, Eva Breuer, Anurag Gupta, Kuno Lehmann

**Affiliations:** 1 Surgical Oncology Research Laboratory, Department of Surgery & Transplantation, University Hospital of Zurich, Raemistrasse 100, 8091 Zurich, Switzerland; lilian.roth@usz.ch (L.R.); Linda.russo@usz.ch (L.R.); Sima.ulugoel@usz.ch (S.U.); rafael.freiredossantos@uzh.ch (R.F.d.S.); Eva.breuer@usz.ch (E.B.); Anurag.gupta@usz.ch (A.G.); 2 University of Zurich, 8006 Zurich, Switzerland

**Keywords:** peritoneal metastasis, colorectal cancer (CRC), cytoreductive surgery, hyperthermic intraperitoneal chemotherapy, tumor biology

## Abstract

**Simple Summary:**

Surgical and locoregional treatments of peritoneal metastasis, e.g., from colorectal cancer, has gained increasing acceptance after the publication of excellent patient outcomes from many groups around the world. Apart from systemic chemotherapy and surgical removal of the tumor, locoregional therapies such as HIPEC or PIPAC may improve tumor control. Understanding the molecular characteristics of peritoneal metastasis is crucial to evolve future therapeutic strategies for peritoneal metastasis. This includes the genetic background of PM, which is often different from other sites of metastasis, and promotes peritoneal dissemination and the growth of tumor cells. Growing knowledge and insight into the physiology of the peritoneal tumor microenvironment and the specific role of the immune system in this compartment may provide a critical step to move locoregional therapy to the next level. This review summarizes the current knowledge and highlights the molecular characteristics of peritoneal metastasis.

**Abstract:**

Peritoneal metastasis (PM) originating from gastrointestinal cancer was considered a terminal disease until recently. The advent of better systemic treatment, a better understanding of prognostic factors, and finally, the advent of novel loco-regional therapies, has opened the door for the multimodal treatment of PM. These strategies, including radical surgery and hyperthermic intraperitoneal chemotherapy (HIPEC) showed surprisingly good results, leading to the prolonged survival of patients with peritoneal metastasis. This has triggered a significant body of research, leading to the molecular characterization of PM, which may further help in the development of novel treatments. This review summarizes current evidence on peritoneal metastasis and explores potential novel mechanisms and therapeutic approaches to treat patients with peritoneal metastasis.

## 1. Introduction

Peritoneal metastasis (PM) often arises from colorectal cancer (CRC). In a recent report, epidemiological patterns of colorectal metastasis were well characterized, revealing that almost one-third of CRC patients have metastatic lesions in the liver, where it often occurs as a single site metastasis. In contrast, about 20% of CRC patients have metastasis in the peritoneum, which often occurs together with other sites, and is associated with mucinous histology [1]. About half of patients with PM present with synchronous disease, the others with metachronous metastasis [2]. The risk to develop metachronous PM was higher in right-sided colon cancer, advanced tumor stage T4, advanced nodal stage N2, as well as in emergency surgery or non-radical resection of the primary tumor [2].

A landmark French study, the so-called EVOCAPE-1 study, prospectively explored the outcome of patients with gastrointestinal PM. This study showed that the median overall survival of patients with PM is dismal, with 3.1 months [3]. Recent studies have presented a benefit of up to 51 months median overall survival of patients with PM from CRC after CRS and HIPEC [4]. This dramatic difference in overall survival is the result of treatment evolution and a better patient selection. 

An important study from the United States compared outcomes of patients with different sites of metastasis under palliative treatment [5]. This study, including a large number of patients, showed an inferior outcome of patients with PM compared with hematogenous metastatic sites. However, this study lacked adequate intraoperative staging and visual assessment of the PCI. This drawback has been addressed in a recent study which compared the outcomes of patients after radical systemic and local treatment for PM, according to sites of recurrent disease [4]. This multicenter study was able to demonstrate an impaired outcome in patients with peritoneal recurrence when compared with hematogenous recurrence. In addition, the authors also observed a different molecular background for peritoneal lesions, which might be a driver for both the site of recurrence and worse outcomes. 

## 2. Genetic Alterations Associated with PM

Over the last decade, genetic analysis of human cancer has evolved enormously, promoting better tumor classifications, improving treatment decisions, and finally enabling personalized treatment strategies. Specific genetic alterations and mutations in PM largely remain unknown. However, it can be assumed that mutations associated with primary tumors may offer a molecular understanding of PM. PM often arises from colorectal cancer, for which genetic understanding has been well characterized [6]. It is known that in CRC, at least seven mutations occur during adenoma-to-carcinoma transformation [7]. In CRC, the loss of APC, mutations in TP53, KRAS, TGF-β and PIK3CA genes, and loss of the chromosome arm 18q are well characterized [8]. Deep molecular assessment has revealed that malignant transformations involve additional steps, e.g., alterations in the promotor region of CpG island tandem repeats of mismatch repair genes such as MLH1, MSH2, MSH6, PMS2, STK11 and SMAD2/4 were identified to cause epigenetic silencing of these genes. Such alterations cause chromosomal instability (CIN), which is common in 65–70% of sporadic CRC [8,9]. CIN can also be promoted by the mutations of genes involved in chromosome segregation (BUB1, AURKA, PLK1, etc.), telomere regulation (TERC) and DNA damage response (ATM, ATR and BRCA1/2). CIN leads to aneuploidy, subchromosomal gene amplification and a high frequency of loss of heterozygosity, promoting metastatic spread [8]. In contrast, microsatellite instability (MSI), resulting from a defect in mismatch repair genes, can cause a further increase in metastatic potential [9]. 

Following molecular assessments such as genetic mutations and MSI status, it is possible to categorize CRC into biologically different tumor types that may also predict clinical prognosis [10]. However, this classification, into the four consensual molecular subtypes, has not yet fully been developed into clinical practice and decision making for targeted therapies or immune therapies, with the exception of the MSI status, which is an accepted selection criterion for checkpoint inhibition. Together with an increasing body of molecular insights into the pathogenesis of CRC, our perception of metastatic diseases is constantly evolving. Currently, a handful of specific genetic alterations are recognized as an underlying mechanism, or at least a hallmark, of a specific metastatic site. For example, differences in APC, BRAF, KRAS and NRAS are associated with the location of the primary tumor in right or left hemicolon [11]. Among them, mutations in KRAS and BRAF were associated with worse overall survival and the site of recurrence in patients with PM [4,12].

## 3. Molecular Steps from Primary to Peritoneal Metastasis

The metastatic cascades of CRC that follow PM development show similarities with other types of metastases. It has been proposed that it may start with the exfoliation of single cells or tumor clumps and, in some cases, via tumor spheroids with inverted polarity (TSIPs) [13]. Exfoliation is initiated upon the downregulation of different adhesion molecules at the cell surface, mostly involving E-cadherin [14]. Another factor that may be involved in exfoliation is CNN3, a potential metastasis marker [15]. CNN3 is expressed in different cell types where it binds to actin, calmodulin, troponin C and tropomyosin, and is involved in the regulation and modulation of smooth muscle contraction. Its role in other cells is not yet identified; however, it may play a role in cell motility and invasiveness, because knockdown of this gene results in the decreased invasiveness of metastatic cancer cell lines. In addition, CNN3 may contribute to the initial dissemination of tumor cells by downregulating the adhesion molecule E-cadherin in the primary tumor [15]. Exfoliation is then followed by epithelial to mesenchyme transition (EMT), where cancer cells gain mesenchymal properties, e.g., the upregulation of N-cadherin, loss of cell polarity and vimentin expression. This cadherin switch is also essential during the formation of peritoneal metastasis from serous ovarian cancer [16]. Cancer cells acquire motility, become invasive and increase the resistance to apoptosis [17]. Moreover, it was shown that in solid tumors such as CRC, high intestinal fluid pressure may contribute to spontaneous tumor cell shedding, supporting cancer cell distribution in the surrounding lymph nodes [18]. 

In a next step, via transmesothelial or translymphatic dissemination, free CRC cells can attach to the distant peritoneum. During transmesothelial dissemination, cancer cells adhere directly to the mesothelium, which consists of a monolayer of mesothelial cells supported by a thin basement membrane. Below the basement membrane is the submesothelium, which harbors cellular (fibroblast, macrophages, endothelial cells, etc.) and acellular (collagen, glycoproteins, proteoglycans, etc.) components [14,19]. Adhesion molecules of the immunoglobulin superfamily, such as ICAM-1, PECAM-1 and VCAM-1, are known to favor mesothelial–cancer interactions. Furthermore, pro-inflammatory cytokines such as TNF-α, IL-1β, IL-6 and INF-γ can promote the expression of the immunoglobulin superfamily and induce the contraction of mesothelial cells, exposing the basement membrane to facilitate invasion. In addition to adhesion molecules, the glycosaminglycan hyaluronan can enhance mesothelial–cancer interactions. The mesothelial cells secrete hyaluronan, which binds to the CD44 receptor on cancer cells [14,15]. Moreover, it has been shown that the CD44v6 isoform in patients with stage II/III CRC show poor prognosis in terms of overall survival and disease-free survival [20]. Mechanistically, isoform CD44v6 in CRC inversely correlates with E-cadherin expression and exhibits a positive correlation with vimentin. Detached cancer cells may also metastasize using translymphatic route. To access the submesothelium, cells pass through lymphatic vessels, which normally serve as drainage channels for the serous cavities and as migration passages for macrophages. Similar to ascites and infectious components, cancer cells use lymphatic vessels to accumulate in milky spots. Milky spots are macrophage-rich aggregates. Milky spots in the murine omentum seem to possess a pronounced vascular microenvironment that may favor the early survival of cancer cells [21] via VEGF and CD105 expression [21]. Once cancer cells attach to the mesothelium, they invade into the subperitoneal space either at areas of peritoneal discontinuity or by actively altering the monolayer. Discontinuity occurs due to prior peritoneal injury, for example, via surgery or pro-inflammatory cytokines, which induce the rounding of mesothelial cells resulting in the exposure of the basement membrane [14]. 

An active alteration of the monolayer involves tumor-cell-induced mesothelial apoptosis via the death ligand/receptor system Fas Ligand/Fas [22]. Furthermore, Yao et al. suggested the role of HGF/c-met pathway in patients with CRC metastases. In this study, the authors found an increased expression of c-Met in metastases compared with the primary CRC tumor and a positive correlation of c-Met expression with tumor stages [23]. However, those findings were observed in CRC liver metastases. Thus, the role of c-Met in peritoneal metastases from CRC remains to be explored. 

Once in the sub-peritoneal space, cancer cells and the surrounding stromata cells secrete matrix metalloproteinase (MMP), affecting the extra cellular matrix (ECM), and allowing invasion and migration. High expression of MMP-1, -2, -7, -9 and -13 was shown to correlate with worse outcomes [24]. Tissue inhibitors of metalloproteinase (TIMPs) are known to inhibit the proteolytic activity of MMPs; however, increased TIMP-1 and TIMP-2 concentrations in serum seems to be associated with worse prognosis [25,26]. After successful dissemination in metastatic sites, cancer cells stimulate sustained proliferation via the secretion of growth factors in the surrounding tumor-associated stroma. For example, insulin-like growth factor 1 (IGF-1) was found to be overexpressed in PM from CRC [26]. Additionally, increased plasma levels of IGF-binding protein 3 (IGFBP3), an endogenous antagonist of IGF-1, was found to correlate with lower progression and an improved treatment response [27]. Furthermore, cancer cells release HIF-1α, which regulates the production of VEGF, to access nutrients and oxygen. A study performed by Varghese et al. on 20 metastatic tumors from patient with colorectal adenocarcinoma indicated that peritoneal metastases overexpressed HIF-1α, TIMP-2 and IGF-1 compared with other metastatic sites [26]. 

Taken together, the induction of peritoneal metastasis involves a complex cascade, starting with tumor cells exfoliation, epithelial-to-mesenchyme transition, attachment, and invasion towards deeper layers by a transmesothelial or translymphatic route; thus, it can be summarized as peritoneal metastatic cascade [14]. The above-mentioned mechanisms are driven mainly by the biology of the tumor cells; however, there is another major player controlling malignant disease: the immune system. 

## 4. Immune Reactions towards Peritoneal Metastasis

More than 100 years ago, in 1909, Paul Ehrlich suggested that the immune system may control tumor development [28]. Almost 50 years after Ehrlich, Brunet and Thomas made an unproven claim that lymphocytes can eliminate transformed cancerous cells. The most elegant experiments regarding the immunosurveillance of cancer came from Robert Schreiber, who introduced the 3E concepts of immunoediting during cancer development [28,29]. The 3Es stand for elimination, equilibrium and escape. In the elimination phase, which is difficult to study experimentally, it is assumed that aberrant cells are actively removed by the immune system. Escape from elimination allows transformed cells to remain in an equilibrium phase, where transformed cells remain on watch and are actively controlled by the immune system, keeping them in a dormant state for many years. However, during this dynamic equilibrium phase, it may happen that cancer cells develop strategies making immune recognition futile. As soon as immune inhibitory changes occur, cancer cells enter the escape phase, leading to the development of clinically apparent cancers [28,29].

The immunoediting process illustrates that if the immune system can be reprogramed to recognize cancer, it will be possible to regain control overgrowth of cancer or metastatic lesions. Many research projects over the past two decades have generated a solid body of evidence indicating that the presence of lymphocytes, especially functional CD8+ T cells, is crucial to control tumor growth. This finding has promoted the evolution of immunotherapies, either involving the transfusion of antigen-specific CD8+ T cells, so called CAR T cells, or antibodies that allow the sustained activation of functional CD8+ T cells. The antibodies target so-called checkpoint blockade molecules such as PD-1, LAG-3, PD-L1 and CTLA-4 [30], and are only effective if sufficient numbers of CD8+ T cells are present within immunogenic tumors. The progress made in the treatment of metastatic melanoma offering prolonged overall survival for a majority of patients illustrates the success of immunotherapy. Unfortunately, with the exception of microsatellite-instable (MSI) colon cancer, immunotherapy thus far has demonstrated limited effects against colorectal cancer (CRC). 

Tumor microenvironment composition is a prognostic factor for overall and cancer-specific survival, regardless of the lymph node, MSI and CpG island methylation status [31]. One important molecular phenotype of CRC is the sporadic or hereditary (Lynch Syndrome) defect in mismatch repair proteins, which leads to MSI. Approximately 15% of all CRCs harbor a microsatellite instability and are associated with a better prognosis [32,33]. The defect in the mismatch repair machinery is either sporadic, caused by the gene silencing of MLH 1, or by a germline mutation of MLH 1 and 2 in Lynch Syndrome. Colon cancer with an MSI phenotype shows a presence of more neoantigens and a higher degree of tumor-infiltrating lymphocytes [34,35] leading to a better immune-recognition, and finally, improved CRC-specific survival [35]. 

In the context of peritoneal metastasis, the role of the immune system in controlling PM development remains unexplored. From liver metastasis, we have learned that cytotoxic (CD8+) T cells seem to play a role. Liver metastasis from colon cancer is associated with a higher CD8/CD3 ratio than liver metastasis from rectal cancer, and has a better progression-free and overall survival [36]. Compared with primary colon cancer, the PM microenvironment shows more NK cell infiltration and more senescent tumor cells [37]. Upon the analysis of *n* = 43 PM samples from gastric cancer, a higher accumulation of resting CD4+ T cells in an aggressive gastric tumor phenotype was found. In contrast, the numbers of cytotoxic T cells, NK cells and myeloid dendritic cells were lower in advanced (G2 vs. G3) and histologically aggressive stages (signet ring vs. non-signet ring). This can be interpreted as an immunosuppressive tumor microenvironment, induced by cancer cells, to facilitate tumor progression. Furthermore, based on the T cell infiltration profile, patients could be divided into T-cell-exclusive and T-cell-exhausted. The T-cell-exclusive group exhibited a low expression of CD8+-T-cell-related cytokines and the immune checkpoint TIM-3, whereas the T-cell-exhausted immune cell infiltrate was characterized by a high expression of cytolytic markers and TIM-3 [38]. 

The role for CD8+-mediated immunity in peritoneal metastasis from CRC remains elusive. However, some evidence is available from patients with epithelial ovarian cancer, where a higher intra-tumoral CD8+ T cell/Tregs ratio was also associated with a prolonged survival [39]. This observation corelates with the clinical observation of tumor dissemination in high-grade serous ovarian cancer, where one group proposed a new classification of peritoneal spread into miliary and non-miliary cancer [40]. A non-miliary pattern of peritoneal metastasis was associated with a better survival regardless of clinicopathological factors, due to more CD8+ T cells with a higher expression of PD-1 and PD-L1 on tumor cells, indicating an activated specific immune response, whereas the miliary form was associated with signs of a systemic inflammation [41]. In addition, PD-L1 expression on primary ovarian cancer is 66% concordant with its peritoneal metastasis, regardless of any significant correlation with the overall survival, progression-free survival and disease-specific survival [42].

## 5. CRS and HIPEC, a Locoregional Treatment Approach for Peritoneal Metastasis 

A few decades ago, patients with PM from colorectal cancer (CRC) had a poor prognosis and seemed incurable [43]. Over recent years, the management and treatment of PM has undergone major developments [44]. The introduction of cytoreductive surgery (CRS), together with hyperthermic intraperitoneal chemotherapy (HIPEC), gave hope to patients with PM [45,46]. During CRS/HIPEC, to eradicate residual microscopic cancer cells, a heated solution with chemotherapeutic agents is applied perioperative directly into the peritoneum after macroscopically visible tumor nodules have been surgically removed. Historically, extensive debulking surgery started in around 1930 by J.V. Meigs for ovarian cancers, and the importance of cytoreductive surgery and, subsequently, of adjuvant chemotherapy became more evident over the next decades, not only for ovarian cancer, but also for pseudomyxoma peritonei [47,48,49,50]. With the increasing use of adjuvant chemotherapy, the idea of intraperitoneal application emerged to increase the therapeutic index [51]. In the following years, several trials with CRS/HIPEC and optimization attempts with different chemotherapeutic agents, depending on histological origin, were conducted [44]. To date, two main surgical intraperitoneal delivery techniques of hyperthermic intraperitoneal chemotherapy have been described: the open coliseum technique and closed administration [52]. In the 1990s, Sugarbaker and his team proposed the peritoneal cancer index (PCI) as a standardized scoring system to quantify peritoneal tumor burden during an operation, and the completeness of cytoreduction score (CC score) to determine how radical the surgery was [53]. The introduction of these scores is relevant for the selection of patients for CRS/HIPEC or to compare results among centers [54]. The contemporary staging of PM is performed with the peritoneal cancer index (PCI), a staging system ranging from 0 to 39 points, which adequately describes the distribution and the size of PM in a patient [55]. The comparison of PM with other metastatic sites is often problematic. Imaging modalities (e.g., computed tomography or magnetic resonance imaging) are more sensitive for metastasis in the lung or liver. Nevertheless, a review article on imaging diagnoses of PM from gastrointestinal and ovarian cancer showed an adequate pooled sensitivity and specificity of 80% and 90%, respectively, for PET-CT, and 92% and 85% for MRI. The authors claim that MRI could become the imaging modality of choice in staging PM, because MRI is already widely available [56]. In a clinical trial (NCT03314649), the role of mutated DNA of cancer cells in the abdominal cavity of gastric cancer patients is assessed, with the goal of increasing sensitivity in the diagnosis of micrometastasis. Another interesting approach is the detection of peritoneal implants from CRC with near-infrared fluorescence imaging after the i.v. administration of indocyane green (ICG) (NCT02032485). Even though many new strategies in the diagnostic procedure are currently being investigated, current clinical practice still relies on the surgical staging of PM by either laparoscopy or laparotomy [57]. 

Although the surgical concepts for cytoreduction are standardized, no standardization has been established thus far for HIPEC protocols with regard to temperature, treatment duration or the type of drug. Current HIPEC protocols for patients with colorectal PM therefore lack consistency and differ among countries and hospitals. For example, one proposed treatment protocol is the use of heated Oxaliplatin (Oxa), a third-generation platinum forming intra- and interstrand crosslinks in the DNA, for 30 min at 43 °C [58]. This protocol was also used in a prospective multicenter trial, PRODIGE-7. Patients (*n* = 265) with stage IV colorectal cancer with isolated peritoneal metastases and a PCI < 25 were randomly assigned to CRS or CRS with an oxaliplatin-based HIPEC for 30 min. No significant benefit regarding median overall survival could be shown by the addition of HIPEC (median OS 41.7 versus 41.2 months). Despite this result, the subgroup of patients with a PCI > 11 ≤ 15 demonstrated a significantly higher overall survival after CRS/HIPEC compared with CRS only [59]. The efficacy of HIPEC in general, with other drug combination, or at other concentrations cannot be answered by this study. Based on the results from both treatment groups, the main message of PRODIGE7 is that CRS, performed in expert centers, provides superior outcomes in patients with PM from CRC. The optimal regimen for HIPEC and the added benefit remains elusive [59].

This result goes well with the observation from a previously completed, small (*n* = 105) randomized trial comparing systemic chemotherapy with CRS/HIPEC [60]. Despite a weak control arm, where patients received only 5-Fluorouracil (5-FU)-based systemic therapy, the authors were able to show that CRS/HIPEC improved disease-specific survival from 12.6 months in the control arm to 22.2 months in the CRS/HIPEC [61]. In both trials, patients who profited had a high peritoneal disease load. 

In 2013, the American Society of Peritoneal Surface Malignancies recommended a regimen with Mitomycin C (MMC) for 90 min at 42 °C, which is now used widely [62,63]. MMC is an antitumor antibiotic, isolated from Streptomyces caespitosus, classified as an alkylating agent causing cross-linking in the DNA. These crosslinks prevent the separation of complementary DNA strands, inhibiting DNA replication [64]. The American randomized phase II study ICARuS with HIPEC using MMC is currently ongoing (NCT01815359), and the adjuvant HIPECT 4 trial is enrolling patients to investigate the role of adjuvant chemotherapy with MMC in order to prevent PM recurrence [65]. Apart from mono regimens with oxaliplatin or mitomycin C, some groups use combination therapies with mitomycinC/doxorubicin or irinotecan (IRI)/doxorubicin [63], although clinical trials with combination therapies are still lacking. 

As mentioned above, patient selection for CRS/HIPEC is key. Negative predictive factors after CRS/HIPEC are the extent of the disease (PCI), nodal stage, tumor biology, response systemic therapy or major complications after CRS/HIPEC. Some of these prognostic factors can be summarized by clinical scores (e.g., PDSS or BIOSCOPE). For example, BIOSCOPE includes the PCI, the nodal stage, tumor grading and the RAS/RAF mutational status, and helps to discriminate prognostic groups [66]. In clinical practice, these scores are usually not exclusive, but may help in the clinical decision process. After CRS/HIPEC, a major complication (Clavien-Dindo classification IIIB or higher) rate of 8.3% to 24% has been published in recent series [67,68].

HIPEC can also be performed as an adjuvant treatment to prevent peritoneal spread. In their trial, Virzi et al. demonstrated the feasibility and safety of HIPEC in an adjuvant setting, even though 16% of patients experienced major complications [69]. The COLOPEC trial could not prove any benefit in peritoneal-free survival after adjuvant HIPEC compared with the control arm [70].

## 6. The Rapid Development of Systemic Chemotherapy

Since the use of 5FU, the field of medical oncology has evolved dramatically and new systemic regimens and targeted agents have significantly improved the survival of patients with advanced stages of CRC [71]. Today, the choice of a systemic chemotherapy regimen depends decisively on the molecular pathological profile of the tumor. Frequently used systemic first-line regimens include combinations of 5FU or capecitabine with oxaliplatin or irinotecan [18]. Triple therapy with 5FU, oxaliplatin and irinotecan (FOLFOXIR) is a very effective regimen and has demonstrated superior response rates [72]. However, due to its side effects, it is usually given to patients in a good health condition [73]. Targeted therapies, e.g., cetuximab, an EGFR-antibody, or bevacizumab, a humanized IgG monoclonal antibody targeting VEGF-A, are often added, depending on the molecular profile of the tumor. Multiple trials, including the phase III CRYSTAL and PRIME study, showed that adding an anti-EGFR to FOLFIRI and FOLFOX4, respectively, is beneficial for patients with RAS wild-type tumors [20]. Overall, median overall survival has dramatically improved to nearly 30 months in the context of aggressive chemotherapy, which often enables secondary surgery of metastasis [72] (Table 1). The majority of patients included in these trials, however, have hematogenous metastasis, which has a significantly better prognosis than peritoneal metastasis [4,5]. Nevertheless, response to neoadjuvant systemic treatment is also a prognostic factor in patients with PM [74]. Thus, progress in this field is likely to enable more aggressive surgery in the future. 

## 7. Novel Concepts and New Treatment Strategies for the Treatment of PM

Apart from using HIPEC as a complementary treatment, directly after cytoreductive surgery, some novel concepts have been introduced. Many of them highlight the palliative aspect of PM. A good example is pressurized intraperitoneal aerosol chemotherapy (PIPAC), where low-dose chemotherapy is applied intraperitoneally during laparoscopy as an aerosol at a pressure of 12 mmHg, usually every four weeks. This method is currently used in patients with non-resectable disease and is well tolerated [78]. The rationale for PIPAC is a more homogeneous intraperitoneal distribution of chemotherapy in a closed space by applying it as a pressurized aerosol, leading to increased chemotherapy concentrations within the tissue compared with HIPEC [77,78]. PIPAC also offers a possibility to repeat the treatment. A further development of PIPAC is electrostatic PIPAC (ePIPAC). Here, a phase II study (NCT03246321) has completed patient recruitment using ePIPAC with oxaliplatin as a palliative monotherapy for patients with isolated and unresectable PM-CRC.

There are multiple challenges in the diagnosis and treatment of peritoneal metastasis. A major issue is the detection of minimal disease during surgery, where scars from previous procedures are impossible to differentiate from metastatic lesions. Techniques such as fluorescent-guided surgery could amplify detection sensitivity, making resections more precise. Several therapeutic targets have being tested; among them, anti-CEA antibodies have shown encouraging clinical results [79]. Another field of research is the delivery of drugs into the peritoneal cavity. Here, apart from HIPEC or PIPAC, several technologies have been investigated that may improve the exposure of PM lesions to cytostatic drugs. For example, nanoparticles could provide several advantages, e.g., prolonged drug retention time, controlled drug release, and serve as a versatile vector for different molecules [80]. The combined used of microspheres and hyaluronic acid hydrogel showed good results, enhanced drug solubility, prolonged drug release and sustained biocompatibility [81]. Another innovative concept is NIPS, where a intraperitoneal catheter is placed into the pelvic cavity to administer neoadjuvant intraperitoneal and systemic chemotherapy. In patients with peritoneal metastasis from gastric cancer, NIPS resulted in a lower cancer-cell-positive ascites rate and in a higher R0 resection rate [82].

Given the increasing knowledge on anti-tumor immunity in the peritoneum, immune checkpoint inhibitors may play a role, not only in micro-satellite instability-high (MSI-H), or mismatch repair-deficient (MMRd) metastatic colorectal or ovarian cancer [83,84,85]. Although the immune cell landscape in PM lesions is not yet established, locoregional treatment may influence immunity in PM lesions. For example, anthracyclines—such as doxycycline—induce immunogenic cell death (ICD) [86], which leads to the release of DAMPs from dying cancer cells. DAMPs in combination with antigen release can result in DC maturation and antigen presentation to CD8+ T cells [86,87]. After recognizing a specific antigen, T cells clonally expand and can fight surviving cancer cells. Moreover, cisplatin (also used for HIPEC) sensitized spontaneous lung cancer in a mouse model to induce a checkpoint blockade via the induction of ICD. This immunogenic effect is mediated not only by chemotherapy, but also radiotherapy; a single carefully selected dose is able to mediate similar effects. Sharma et al. described an enhanced expression of cancer testis antigens and MHC-I expression after the application of 20 Gy radiotherapy in vitro [88]. Taken together, locoregional therapy may induce ICD to activate the immune system. In the context of HIPEC, the addition of hyperthermia might have an additional effect through the induction of heat shock proteins. In mice, an Hsp-90-mediated anticancer immune response was observed after the in vitro HIPEC treatment of murine cancer cells [89]. An interesting example for a novel treatment approach for PM-CRC was provided by the phase 1 ImmunoPeCa trial. The immunotoxin MOC31PE was intraperitoneally administered one day after CRS and HIPEC, with the intention of killing EpCAM-positive cancer cells and preventing recurrence [90]. 

**Figure 1:**
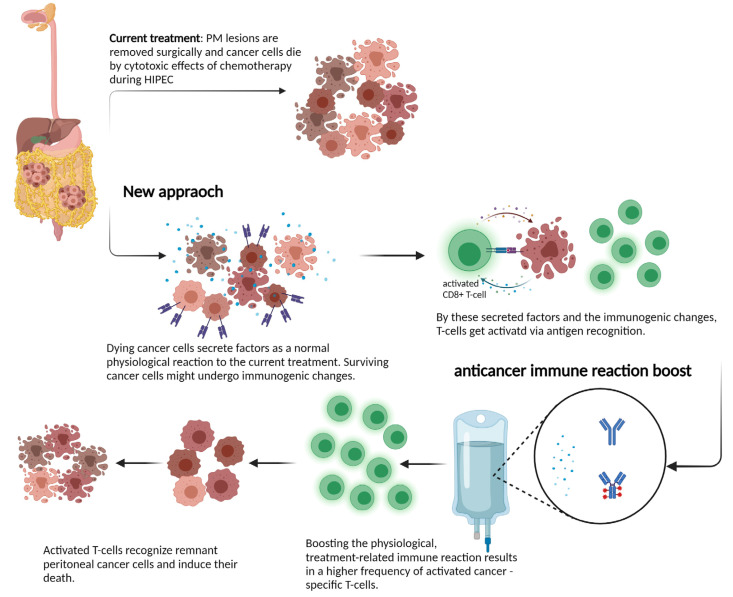
Schematic view of mechanisms of locoregional treatment in the peritoneum: the current interpretation of how HIPEC or PIPAC act on tumor cells is to induce cell death via direct cytotoxicity. Growing evidence on immunogenic cell death suggests that the activation of a patient’s immune system might mediate better long-term disease control through the induction of T cells. This process might be boosted in next-generation treatment approaches and induce a profound and sustained immune reaction against metastatic lesions. Created with BioRender.com.

Despite the recent progress made, a deeper insight and molecular understanding of effects during locoregional treatment on the human host physiology and the immune system is required. Investigating tumor samples from PM patients and further assessments of cytokine profiles before and after treatment could provide critical knowledge about these processes. If surgery and locoregional treatment can induce anti-tumor immunity, these patients might profit from an additional treatment to boost the immune reaction. The rationale behind such a novel treatment approach is shown in Figure 1. 

## 8. Conclusions and Outlook

Understanding the molecular characteristics of peritoneal metastasis is crucial to evolve future therapeutic strategies for peritoneal metastasis. This includes the genetic background of PM, which is often different from other sites of metastasis, and promotes peritoneal dissemination and the growth of tumor cells. Growing knowledge and insight into the physiology of the peritoneal tumor microenvironment and the specific role of the immune system in this compartment may provide a critical step to move locoregional therapy to the next level. 

It is likely that progress made in systemic treatment regimens will translate into better response rates for peritoneal metastasis and enable radical treatment in more patients. Better knowledge on selection criteria will help to identify patients who are likely to profit from aggressive surgical treatment, whereas patients with an adverse risk profile may undergo additional locoregional treatment, e.g., with PIPAC. Finally, optimal drug regimens for the locoregional treatment of PM remain unclear, and the identification of molecular mechanisms involved in long-term tumor control beyond cytotoxicity is critical. Therefore, experimental, and translational research is critical in the next years. 

## Figures and Tables

**Table 1:** Current data on the treatment of PM from CRC by either systemic therapy alone or in combination with locoregional treatment. It is critical to highlight that the amount of disease in the peritoneum or the chemotherapy regimen differed among the studies. (amount of disease: +++ extensive load of PM, ++ moderate load, + limited load, NA: not available).

PM—CRC Outcome after Different Treatment Approaches					
	mOS (Months)				
	CRS/HIPEC	Systemic Chemotherapy	PIPAC	Amount of Disease	Used Drug
Vervaal V. et al., 2003 [60]	22.2	12.6	-	+++	5FU
Elias D. et al., 2009 [58]	67.7	23.9	-	++	FOLFOX/FOLFIRI
Franko J. et al., 2016 [5]	16.3	-	-	NA	FOLFOX/FOLFIRI +/−ab
Cremolini Ch. et al., 2020 [75]	-	28.9	-	NA	FOLFOXIRI
Quenet F. et al., 2021 [59]	41	-	-	++	FOLFOX/FOLFIRI
Breuer E. et al., 2021 [4]	51	-	-	++	FOLFOX/FOLFIRI
Demtröder C. et al., 2015 [76]	-	-	15.7	+++	FOLFOX/FOLFIRI +/−ab
Goére D. et al., 2020 [77]	mOS not reached during 50.8 months of follow-up	-		+	FOLFOX/XELOX

## Abbreviations

CRS	cytoreductive surgery
CRC	colorectal cancer
DFS	disease-free survival
HIPEC	hyperthermic intraperitoneal chemotherapy
mOS	median overall survival
MSI/MSS	microsatellite instability/stability
PM	peritoneal metastasis
PCI	peritoneal cancer index
RCT	randomized controlled trial
